# Associations of height, body mass index, and weight gain with breast cancer risk in carriers of a pathogenic variant in *BRCA1* or *BRCA2*: the *BRCA1* and *BRCA2* Cohort Consortium

**DOI:** 10.1186/s13058-023-01673-w

**Published:** 2023-06-20

**Authors:** Karin Kast, Esther M. John, John L. Hopper, Nadine Andrieu, Catherine Noguès, Emmanuelle Mouret-Fourme, Christine Lasset, Jean-Pierre Fricker, Pascaline Berthet, Véronique Mari, Lucie Salle, Marjanka K. Schmidt, Margreet G. E. M. Ausems, Encarnacion B. Gomez Garcia, Irma van de Beek, Marijke R. Wevers, D. Gareth Evans, Marc Tischkowitz, Fiona Lalloo, Jackie Cook, Louise Izatt, Vishakha Tripathi, Katie Snape, Hannah Musgrave, Saba Sharif, Jennie Murray, EMBRACE Collaborators, Sarah V. Colonna, Irene L. Andrulis, Mary B. Daly, Melissa C. Southey, Miguel de la Hoya, Ana Osorio, Lenka Foretova, Dita Berkova, Anne-Marie Gerdes, Edith Olah, Anna Jakubowska, Christian F. Singer, Yen Tan, Annelie Augustinsson, Johanna Rantala, Jacques Simard, Rita K. Schmutzler, Roger L. Milne, Kelly-Anne Phillips, Mary Beth Terry, David Goldgar, Flora E. van Leeuwen, Thea M. Mooij, Antonis C. Antoniou, Douglas F. Easton, Matti A. Rookus, Christoph Engel

**Affiliations:** 1 Center for Hereditary Breast and Ovarian Cancer, Center for Integrated Oncology (CIO), Medical Faculty, University Hospital Cologne, Kerpener Str. 62, 50937 Cologne, Germany; 2 Department of Epidemiology & Population Health and of Medicine (Oncology), Stanford University School of Medicine, Stanford, CA USA; 3 Stanford Cancer Institute, Stanford University School of Medicine, Stanford, CA USA; 4 Centre for Epidemiology and Biostatistics, Melbourne School of Population and Global Health, The University of Melbourne, Melbourne, VIC Australia; 5 INSERM U900, Paris, France; 6 Institut Curie, Paris, France; 7 Mines Paris Tech, Fontainebleau, France; 8 PSL Research University, Paris, France; 9 Aix Marseille Université, INSERM, IRD, SESSTIM, Marseille, France; 10 Département d’Anticipation et de Suivi Des Cancers, Oncogénétique Clinique, Institut Paoli-Calmettes, Marseille, France; 11 Centre Léon Bérard, Lyon, France; 12 CRLCC Paul Strauss, Strasbourg, France; 13 Centre François Baclesse, Caen, France; 14 Centre Antoine Lacassagne, Nice, France; 15 Oncogénétique Poitou-Charentes, Niort, France; 16 Division of Molecular Pathology, Netherlands Cancer Institute, Antoni Van Leeuwenhoek Hospital, Amsterdam, The Netherlands; 17 Department of Genetics, Division Laboratories, Pharmacy and Biomedical Genetics, University Medical Center Utrecht, Utrecht, The Netherlands; 18 Department of Clinical Genetics, Maastricht University Medical Center, Maastricht, The Netherlands; 19 Department of Human Genetics, Amsterdam UMC, University of Amsterdam, Amsterdam, The Netherlands; 20 Department of Clinical Genetics, Radboud University Medical Center, Nijmegen, The Netherlands; 21 The Prevent Breast Cancer Research Unit, The Nightingale Centre, Manchester University NHS Foundation Trust, Manchester, UK; 22 Genomic Medicine, Division of Evolution and Genomic Sciences, The University of Manchester, St Mary’s Hospital, Manchester University NHS Foundation Trust, Manchester, UK; 23 Manchester Breast Centre, Oglesby Cancer Research Centre, The Christie, University of Manchester, Manchester, UK; 24 Department of Medical Genetics, National Institute for Health Research Cambridge Biomedical Research Centre, University of Cambridge, Cambridge, UK; 25 Clinical Genetics Service, Manchester Centre for Genomic Medicine, Central Manchester University Hospitals NHS Foundation Trust, Manchester, UK; 26 Sheffield Clinical Genetics Service, Sheffield Children’s Hospital, Sheffield, UK; 27 Department of Clinical Genetics, Guy’s and St Thomas’ NHS Foundation Trust, London, UK; 28 Clinical Genetics Service, Guy’s and St Thomas’ NHS Foundation Trust, London, UK; 29 Department of Clinical Genetics, St George’s University Hospitals NHS Foundation Trust, London, UK; 30 Yorkshire Regional Genetics Service, Leeds Teaching Hospitals NHS Trust, Leeds, UK; 31 West Midlands Regional Genetics Service, Birmingham Women’s Hospital Healthcare NHS Trust, Edgbaston, Birmingham, UK; 32 South East of Scotland Regional Genetics Service, Western General Hospital, Edinburgh, UK; 33 MRC Human Genetics Unit, Institute of Genetics and Cancer, University of Edinburgh, Edinburgh, UK; 34 Centre for Cancer Genetic Epidemiology, Department of Public Health and Primary Care, University of Cambridge, Cambridge, UK; 35 Department of Medicine and Huntsman Cancer Institute, University of Utah Health, Salt Lake City, UT USA; 36 Fred A. Litwin Center for Cancer Genetics, Lunenfeld-Tanenbaum Research Institute of Mount Sinai Hospital, Toronto, ON Canada; 37 Department of Molecular Genetics, University of Toronto, Toronto, ON Canada; 38 Department of Clinical Genetics, Fox Chase Cancer Center, Philadelphia, PA USA; 39 Precision Medicine, School of Clinical Sciences at, Monash Health Monash University, Clayton, VIC Australia; 40 Department of Clinical Pathology, The University of Melbourne, Melbourne, VIC Australia; 41 Molecular Oncology Laboratory, Hospital Clínico San Carlos, IdISSC (Instituto de Investigación Sanitaria del Hospital Clínico San Carlos), Madrid, Spain; 42 Familial Cancer Clinical Unit, Human Cancer Genetics Programme, Spanish National Cancer Research Centre (CNIO) and Spanish Network On Rare Diseases (CIBERER), Madrid, Spain; 43 Department of Cancer Epidemiology and Genetics, Masaryk Memorial Cancer Institute, Brno, Czech Republic; 44 Department of Clinical Genetics, Rigshospitalet, Copenhagen University Hospital, Copenhagen, Denmark; 45 Department of Molecular Genetics, National Institute of Oncology, Budapest, Hungary; 46 Department of Genetics and Pathology, Pomeranian Medical University, Szczecin, Poland; 47 Independent Laboratory of Molecular Biology and Genetic Diagnostics, Pomeranian Medical University, Szczecin, Poland; 48 Department of OB/GYN and Comprehensive Cancer Center, Medical University of Vienna, Vienna, Austria; 49 Department of Oncology, Clinical Sciences in Lund, Lund University Hospital, Lund, Sweden; 50 Clinical Genetics, Karolinska Institutet, Stockholm, Sweden; 51 Genomics Center, Centre Hospitalier Universitaire de Québec-Université Laval Research Center, Quebec City, QC Canada; 52 Cancer Epidemiology Division, Cancer Council Victoria, Melbourne, VIC Australia; 53 Precision Medicine, School of Clinical Sciences at Monash Health, Monash University, Clayton, VIC Australia; 54 The Sir Peter MacCallum Department of Oncology, University of Melbourne, Parkville, Australia; 55 Department of Medical Oncology, Peter MacCallum Cancer Centre, Victoria, Australia; 56 Department of Epidemiology, Mailman School of Public Health and the Herbert Irving Comprehensive Cancer Center, Columbia University Irving Medical Center, New York, NY USA; 57 Department of Dermatology, University of Utah School of Medicine, Salt Lake City, UT USA; 58 Department of Epidemiology, Netherlands Cancer Institute, Amsterdam, The Netherlands; 59 Centre for Cancer Genetic Epidemiology, Department of Oncology, University of Cambridge, Cambridge, UK; 60 Institute for Medical Informatics, Statistics and Epidemiology, Leipzig University, Leipzig, Germany

## Abstract

**Introduction:**

Height, body mass index (BMI), and weight gain are associated with breast cancer risk in the general population. It is unclear whether these associations also exist for carriers of pathogenic variants in the *BRCA1* or *BRCA2* genes.

**Patients and methods:**

An international pooled cohort of 8091 *BRCA1/2* variant carriers was used for retrospective and prospective analyses separately for premenopausal and postmenopausal women. Cox regression was used to estimate breast cancer risk associations with height, BMI, and weight change.

**Results:**

In the retrospective analysis, taller height was associated with risk of premenopausal breast cancer for *BRCA2* variant carriers (HR 1.20 per 10 cm increase, 95% CI 1.04–1.38). Higher young-adult BMI was associated with lower premenopausal breast cancer risk for both *BRCA1* (HR 0.75 per 5 kg/m^2^, 95% CI 0.66–0.84) and *BRCA2* (HR 0.76, 95% CI 0.65–0.89) variant carriers in the retrospective analysis, with consistent, though not statistically significant, findings from the prospective analysis. In the prospective analysis, higher BMI and adult weight gain were associated with higher postmenopausal breast cancer risk for *BRCA1* carriers (HR 1.20 per 5 kg/m^2^, 95% CI 1.02–1.42; and HR 1.10 per 5 kg weight gain, 95% CI 1.01–1.19, respectively).

**Conclusion:**

Anthropometric measures are associated with breast cancer risk for *BRCA1* and *BRCA2* variant carriers, with relative risk estimates that are generally consistent with those for women from the general population.

**Supplementary Information:**

The online version contains supplementary material available at 10.1186/s13058-023-01673-w.

## Introduction

Taller height and higher postmenopausal body mass index (BMI) are well-established risk factors for breast cancer [1-5]. While weight gain in adulthood increases the risk of postmenopausal breast cancer, higher weight in adolescence and early adulthood has been associated with decreased risk of pre- and postmenopausal breast cancer [3, 6-8]. The latter may be due in part to the fact that the differentiation of the mammary glands is promoted under higher estrogen levels in obese adolescents and young adults [4, 9-12].

Carriers of germline pathogenic variants in the *BRCA1* or *BRCA2* breast cancer predisposition genes have high breast cancer risks [13]. However, the extent to which these risks are modified by anthropometric factors is unknown. Most previous studies have been retrospective, some of them with limited sample sizes, and their results have generally been inconclusive [14-21]. Three previous studies examined the association of height with breast cancer risk in pathogenic variant carriers [17, 18, 20]. While two studies reported a positive association between height and breast cancer risk [17, 20], one did not observe an association [18]. These studies also analyzed body weight and breast cancer risk by menopausal status. Manders et al. found an association of higher current weight with postmenopausal breast cancer risk in a cohort of 299 carriers of pathogenic variants in *BRCA1* or *BRCA2* [17]. They also found weak evidence of a positive association between weight gain and postmenopausal breast cancer [17]. The retrospective study by Qian et al. reported an inverse association for higher BMI at age 18 years and risk of premenopausal breast cancer in a cohort of 14,676 carriers of pathogenic variants in *BRCA1* and 7912 in *BRCA2* [20]. Using a Mendelian randomization approach, they additionally showed a similar inverse association between genetically determined BMI and premenopausal breast cancer risk, consistent with that seen in the general population [3, 5-8]. The only prospective study on BMI and breast cancer risk in carriers of a pathogenic variant in *BRCA1* or *BRCA2* found weak evidence for an association of higher BMI at age 18 years with lower postmenopausal breast cancer risk, but no association of current BMI or weight change and breast cancer risk [18].

The identification of non-genetic risk factors for breast cancer in high-risk populations is important for developing more accurate risk prediction models and designing risk-adapted prevention strategies. In the present work, we evaluated the associations of height, BMI, and change in weight with pre- and postmenopausal breast cancer risk for carriers of a pathogenic variant in *BRCA1* or *BRCA2*, using data from the International *BRCA1* and *BRCA2* Cohort Consortium [22-25].

## Methods

### Study sample

We used pooled data from three large prospective cohort studies of *BRCA1* and *BRCA2* pathogenic variant carriers: the International *BRCA1/2* Carrier Cohort Study (IBCCS, consisting of 19 national multi- and single-center prospective cohort studies) [22], the Kathleen Cuningham Foundation Consortium for research into Familial Breast cancer (kConFab) [24, 26], and the Breast Cancer Family Registry (BCFR) [23, 25] (Additional file 1: Table S1). The study sample comprised women who were 18 to 80 years of age at recruitment and tested positive for a pathogenic germline variant in *BRCA1* or *BRCA2.* Women with pathogenic variants in both genes were excluded.

### Data collection

Study participants completed a baseline questionnaire and one or more follow-up questionnaires. The questionnaires asked about risk factors for breast cancer, including height, young-adult weight (age 18 or 20 years), weight at questionnaire completion, reproductive and medical history, surgical interventions, and menstrual history (age at menarche, age at last menstruation, whether the woman had had any periods in the past year, the number of years/months since the last menstruation, and reason(s) for periods stopping) [27].

BMI was calculated as weight (kg) divided by height (m) squared. Weight change was calculated as the difference (in kg) between baseline weight and young-adult weight.

For women who indicated no periods in the past year, age at menopause was determined by adding one year to “age at last menstruation.” Women below the age of 60 years were considered premenopausal if they indicated that they had had a period in the past year, or if the “reason for periods stopping” was medication, oral contraceptive use, pregnancy, or breastfeeding. Women below the age of 60 years reporting risk-reducing salpingo-oophorectomy (RRSO) as the reason for menopause were considered premenopausal until RRSO. For women who reported a hysterectomy without bilateral oophorectomy, menopausal status was considered unknown. For women who were still menstruating when they started hormonal therapy and those who took hormonal contraceptives at older ages, age at menopause was classified as unknown.

Occurrence of breast cancer was derived from follow-up questionnaires and, for some studies, through linkage to cancer registries. Information on vital status was obtained from municipal or death registries, medical records, or family members. For details, see Kuchenbaecker et al. [13]. All study participants provided written informed consent, and each study was approved by the relevant ethics committee at each participating institution.

### Statistical analysis

Associations with breast cancer risk were evaluated using Cox proportional hazards regression with age as the timescale, estimating hazard ratios (HR) and corresponding 95% confidence intervals (CI). Exposure variates analyzed were height, young-adult BMI, baseline BMI, and weight change between early adulthood and baseline. Each anthropometric variable was analyzed in a separate model. All analyses were stratified by year of birth (< 1950, 1950–1959, 1960–1969, ≥ 1970) and study and were adjusted for age at menarche, number of full-term pregnancies, oral hormonal contraceptive use (ever/never), and hormone replacement therapy (ever/never), as reported in the baseline questionnaire. Robust variance estimation was used to account for familial clustering of study participants. Associations were examined separately for retrospective and prospective observation times, i.e., before and after baseline questionnaire, respectively, separately for *BRCA1* and *BRCA2* pathogenic variant carriers, and separately for pre- and postmenopausal women, resulting in eight sets of analyses.

### Retrospective analysis

Associations of breast cancer risk with height and young-adult BMI were analyzed. For premenopausal women, the observation time started at birth and ended at diagnosis of the first primary breast cancer (invasive or in situ) as the event of interest, or was censored at diagnosis of any other type of cancer, risk-reducing mastectomy (RRM), completion of baseline questionnaire, or menopause, whichever came first. For postmenopausal women, observation started at menopause (if menopause occurred before baseline) and was censored at diagnosis of the first primary breast cancer (event), or at diagnosis of any other type of cancer, RRM, or baseline, whichever came first. Due to the non-random sampling of prevalent breast cancer cases, retrospective analyses were performed using the weighted retrospective cohort approach described by Antoniou et al. [28]. Individuals were weighted such that the observed breast cancer incidence rates in the pre- and postmenopausal cohorts were consistent with established age-specific risk estimates for *BRCA1* and *BRCA2* variant carriers [29].

### Prospective analysis

Associations of breast cancer risk with height, young-adult BMI, baseline BMI, and weight change between early adulthood and baseline were analyzed. For premenopausal women, observation time started at baseline questionnaire completion (if women were premenopausal at that time point) and ended at diagnosis of the first primary breast cancer as the event of interest. Observation time was censored at diagnosis of any other type of cancer, RRM, last follow-up, or menopause, whichever came first. For postmenopausal women, observation started at baseline or menopause, whichever came last, and ended at diagnosis of the first primary breast cancer, or at diagnosis of any other type of cancer, RRM, or last follow-up, whichever came first.

If a woman’s prospective observation period had included both pre- and postmenopausal periods, then follow-up periods were assigned to the pre- and postmenopausal analyses, as appropriate.

All reported *P* values are two-sided. *P* values < 0.05 were considered statistically significant. Statistical analyses were conducted using R 4.1.1 for Windows (R Core Team. R: A language and environment for statistical computing. R Foundation for Statistical Computing, Vienna, Austria. URL: https://www.R-project.org) and IBM SPSS Statistics for Windows Version 26 (IBM Corp, Armonk, NY)*.*

## Results

### Retrospective analysis

The retrospective analysis included 6858 pathogenic variant carriers (*BRCA1*: 4257; *BRCA2*: 2601). The mean age at the end of observation was 40.5 years (SD ± 10.6), see Table 1.

**Table 1:** Characteristics of women in the retrospective analysis, by menopausal status

Menopausal status	Premenopausal						Postmenopausal						Pre-/postmenopausal
BRCA status	*BRCA1*			*BRCA2*			*BRCA1*			*BRCA2*			*BRCA1/2*
Breast cancer	Unaffected	Affected	Total	Unaffected	Affected	Total	Unaffected	Affected	Total	Unaffected	Affected	Total	Total
Number	n = 2729	n = 1528	n = 4257	n = 1765	n = 836	n = 2601	n = 451	n = 199	n = 650	n = 327	n = 179	n = 506	n = 6858
Age at Censoring	38.2 ± 9.0	37.5 ± 6.7	37.9 ± 8.2	39.9 ± 8.9	39.9 ± 6.6	39.9 ± 8.2	53.4 ± 9.0	54.2 ± 8.0	53.7 ± 8.7	57.4 ± 8.8	56.0 ± 7.2	56.9 ± 8.3	40.5 ± 10.6
*Year of Birth*, no (%)													
< 1950	581 (21.3)	332 (21.7)	913 (21.4)	465 (26.3)	182 (21.8)	647 (24.9)	218 (48.3)	150 (75.4)	368 (56.6)	205 (62.7)	136 (76.0)	341 (67.4)	1560 (22.7)
1950–1959	552 (20.2)	529 (34.6)	1081 (25.4)	343 (19.4)	295 (35.3)	638 (24.5)	148 (32.8)	39 (19.6)	187 (28.8)	88 (26.9)	41 (22.9)	129 (25.5)	1719 (25.1)
1960–1969	748 (27.4)	487 (31.9)	1235 (29.0)	456 (25.8)	283 (33.9)	739 (28.4)	79 (17.5)	8 (4.0)	87 (13.4)	31 (9.5)	2 (1.1)	33 (6.5)	1974 (28.8)
≥ 1970	848 (31.1)	180 (11.8)	1028 (24.1)	501 (28.4)	76 (9.1)	577 (22.2)	6 (1.3)	2 (1.0)	8 (1.2)	3 (0.9)	0 (0.0)	3 (0.6)	1605 (23.4)
Height (cm)	164.9 ± 6.9	164.6 ± 6.6	164.8 ± 6.8	164.3 ± 6.8	164.6 ± 7.0	164.4 ± 6.8	163.6 ± 6.9	162.1 ± 6.6	163.1 ± 6.8	162.6 ± 6.7	162.3 ± 6.3	162.5 ± 6.5	164.2 ± 6.6
Young adult*													
Weight (kg)	57.5 ± 8.9	56.1 ± 7.9	57.0 ± 8.6	57.9 ± 9.8	56.6 ± 7.9	57.5 ± 9.2	56.8 ± 8.2	56.1 ± 8.3	56.6 ± 8.2	57.9 ± 11.4	55.8 ± 8.5	57.2 ± 10.5	57.2 ± 8.8
BMI (kg/m^2^)	21.1 ± 3.0	20.7 ± 2.6	21.0 ± 2.9	21.4 ± 3.3	20.9 ± 2.6	21.3 ± 3.1	21.2 ± 2.8	21.4 ± 2.9	21.3 ± 2.8	21.9 ± 3.9	21.2 ± 2.9	21.6 ± 3.6	21.1 ± 3.0

Numbers are means ± SD unless otherwise specified

*Age 18 or 20 years

Taller height was associated with higher premenopausal breast cancer risk in *BRCA2* pathogenic variant carriers (HR 1.20 per 10 cm increase, 95% CI 1.04–1.38). A consistent HR estimate was seen among postmenopausal women (HR 1.30 per 10 cm increase, 95% CI 0.97–1.74), see Table 2 and Additional file 2: Table S2. No association was seen for *BRCA1* variant carriers, neither for pre- or menopausal women. Higher young-adult BMI was associated with lower risk of premenopausal breast cancer both in *BRCA1* (HR 0.75 per 5 kg/m^2^, 95% CI 0.66–0.84) and *BRCA2* (HR 0.76 per 5 kg/m^2^, 95% CI 0.65–0.89) variant carriers. Consistent inverse associations with premenopausal breast cancer risk were also seen when young-adult BMI was categorized. In postmenopausal women, high young-adult BMI was associated with reduced breast cancer risk in *BRCA1* carriers compared to women with a normal BMI, although statistical significance was only reached in the category of low compared to normal BMI (< 18.5 kg/m^2^ vs. 18.5 to < 25 kg/m^2^: HR 1.67, 95% CI 1.09–2.56). A consistent, but not statistically significant result was observed for *BRCA2* variant carriers. Young-adult BMI, considered as a continuous variable, was not significantly associated with postmenopausal breast cancer risk.

**Table 2:** Retrospective analysis of height, body mass index, and breast cancer risk, by menopausal status

Menopausal status	Premenopausal								Postmenopausal							
BRCA status	*BRCA1*				*BRCA2*				*BRCA1*				*BRCA2*			
	n	BC	HR	95% CI	n	BC	HR	95% CI	n	BC	HR	95% CI	n	BC	HR	95% CI
Height, per 10 cm	4257	1528	1.06	0.95–1.17	2601	836	1.20	1.04–1.38	650	199	0.87	0.69–1.10	506	179	1.30	0.97–1.74
*Young-adult BMI*, kg/m^2^ (categories)																
< 18.5	714	268	1.20	1.00–1.42	362	132	1.16	0.89–1.51	91	32	1.67	1.09–2.56	63	31	1.58	0.95–2.60
18.5 to < 25 (reference)	3231	1177	1.0		2015	654	1.0		504	148	1.0		394	134	1.0	
≥25	312	83	0.67	0.51–0.88	224	50	0.65	0.46–0.93	55	19	1.10	0.67–1.82	49	14	0.95	0.53–1.69
Young-adult BMI per 5 kg/m^2^ (continuous)	4257	1528	0.75	0.66–0.84	2601	836	0.76	0.65–0.89	650	199	0.86	0.64–1.17	506	179	0.85	0.64–1.14

*BC* breast cancer, *BMI* body mass index, 1.0 = reference value

All analyses were adjusted for age at menarche, number of full-term pregnancies, oral hormonal contraceptive use, and hormone replacement therapy

### Prospective analysis

The prospective analysis was based on 3698 pathogenic variant carriers (*BRCA1*: 2167; *BRCA2*: 1531 with information on height and baseline weight. In total, 397 incident breast cancer cases were diagnosed (Table 3). The premenopausal cohort comprised 2527 carriers (*BRCA1*: 1516; *BRCA2*: 1011), and the postmenopausal cohort 1531 carriers (*BRCA1*: 905; *BRCA2*: 626), with a mean follow-up time of 5.3 ± 3.5 years. Information on young-adult weight was available for a subgroup of 3100 (84%) carriers.

**Table 3:** Characteristics of women in the prospective analysis, by menopausal status

Menopausal status	Premenopausal						Postmenopausal						Pre-/postmenopausal
BRCA status	*BRCA1*			*BRCA2*			*BRCA1*			*BRCA2*			*BRCA1/2*
Breast cancer	Unaffected	Affected	Total	Unaffected	Affected	Total	Unaffected	Affected	Total	Unaffected	Affected	Total	Total
Number	n = 1401	n = 115	n = 1516	n = 960	n = 51	n = 1011	n = 793	n = 112	n = 905	n = 559	n = 67	n = 626	n = 3698
Age at Start	32.4 ± 7.8	33.3 ± 6.0	32.5 ± 7.7	33.9 ± 8.1	37.0 ± 6.4	34.1 ± 8.1	47.8 ± 9.5	48.0 ± 9.0	47.8 ± 9.5	51.4 ± 9.8	52.9 ± 8.4	51.6 (9.7)	38.9 ± 12.0
Age at Censoring	36.7 ± 7.1	37.0 ± 5.5	36.7 ± 7.0	37.8 ± 7.7	39.8 ± 6.0	37.9 ± 7.6	52.7 ± 10.4	51.7 ± 9.4	52.6 ± 10.3	55.9 ± 10.4	55.9 ± 8.8	55.9 ± 10.3	44.0 ± 12.3
Follow–up time (years)	4.3 ± 3.2	3.6 ± 2.9	4.2 ± 3.2	3.9 ± 2.9	2.8 ± 2.5	3.8 ± 2.8	4.9 ± 3.3	3.7 ± 3.1	4.8 ± 3.3	4.5 ± 3.3	3.1 ± 2.7	4.4 ± 3.0	5.2 ± 3.5
*Year of Birth*													
< 1950	11 (0.8)	1 (0.9)	12 (0.8)	7 (0.7)	1 (2.0)	8 (0.8)	184 (23.2)	29 (25.9)	213 (23.5)	185 (33.1)	33 (49.3)	218 (34.8)	449 (12.1)
1950–1959	140 (10.0)	10 (8.7)	150 (9.9)	112 (11.7)	9 (17.6)	121 (12.0)	265 (33.4)	40 (35.7)	305 (33.7)	181 (32.4)	21 (31.3)	202 (32.3)	679 (18.4)
1960–1969	435 (31.0)	55 (47.8)	490 (32.2)	309 (32.2)	27 (52.9)	336 (33.2)	284 (35.8)	41 (36.6)	325 (29.9)	167 (29.9)	13 (19.4)	180 (28.8)	1128 (30.5)
≥ 1970	815 (58,2)	49 (42.6)	864 (57.0)	532 (55.4)	14 (27.5)	546 (54.0)	60 (7.6)	2 (1.8)	62 (4.7)	26 (4.7)	0 (0.0)	26 (4.2)	1442 (39.0)
Height (cm)	165.7 ± 6.8	166.4 ± 7.0	165.7 ± 6.8	165.2 ± 6.6	166.4 ± 6.5	165.3 ± 6.3	164.3 ± 6.8	164.5 ± 7.0	164.3 ± 6.8	163.5 ± 6.8	162.4 ± 6.6	163.4 ± 6.8	164.9 ± 6.9
Weight at baseline (kg)	65.1 ± 13.7	65.2 ± 9.9	65.1 ± 13.4	66.4 ± 13.8	66.5 ± 13.6	66.5 ± 13.8	68.0 ± 14.4	71.0 ± 18.7	68.4 (15)	68.9 ± 15.3	70.6 ± 14.8	69.1 ± 15.2	67.0 ± 14.4
BMI at baseline (kg/m^2^)	23.7 ± 4.8	23.6 ± 3.9	23.7 ± 4.7	24.3 ± 4.9	24.0 ± 4.5	24.3 ± 4.8	25.2 ± 5.2	26.2 ± 6.5	25.3 ± 5.4	25.8 ± 5.5	26.7 ± 5.3	25.9 ± 5.5	24.7 ± 5.2

Breast cancer	Unaffected	Affected	Total	Unaffected	Affected	Total	Unaffected	Affected	Total	Unaffected	Affected	Total	Total
Number	n = 1146	n = 96	n = 1242	n = 789	n = 40	n = 829	n = 640	n = 89	n = 729	n = 453	n = 56	n = 509	n = 3100
Young adult*													
Weight (kg)	58.5 ± 9.9	57.7 ± 9.4	58.5 ± 9.9	59.5 ± 10.1	58.3 ± 8.4	59.5 ± 10.0	56.9 ± 8.7	56.9 ± 9.0	56.9 ± 8.7	57.7 ± 10.6	58.6 ± 12.3	57.8 ± 10.8	58.4 ± 10.0
BMI (kg/m^2^)	21.3 ± 3.4	20.8 ± 3.2	21.3 ± 3.4	21.8 ± 3.4	21.2 ± 2.6	21.7 ± 3.4	21.1 ± 3.0	21.0 ± 2.8	21.1 ± 2.9	21.6 ± 3.7	22.2 ± 4.3	21.7 ± 3.8	21.5 ± 3.4
Weight change total (kg)	6.4 ± 9.8	7.7 ± 10.2	6.5 ± 9.9	7.1 ± 10.5	9.2 ± 10.4	7.2 ± 10.5	11.0 ± 11.3	13.9 ± 15.2	11.3 ± 11.9	11.2 ± 11.8	12.7 ± 14.1	11.4 ± 12.1	8.6 ± 11.1
*Weight change*													
≥ 5 kg loss	87 (7.6)	6 (6.3)	93 (7.5)	50 (6.3)	2 (5.0)	52 (6.3)	24 (3.8)	4 (4.5)	28 (3.8)	23 (5.1)	6 (10.7)	29 (5.7)	190 (6.3)
< 5 kg loss to < 5 kg gain	472 (41.2)	34 (35.4)	506 (40.7)	313 (39.7)	11 (27.5)	324 (39.1)	165 (25.8)	18 (20.2)	183 (25.1)	114 (25.2)	7 (12.5)	121 (23.8)	1035 (34.1)
≥ + 5 kg to 15 kg gain	404 (35.3)	34 (35.4)	438 (35.3)	282 (35.7)	19 (47.5)	301 (36.3)	254 (39.7)	34 (38.2)	288 (39.5)	167 (36.9)	27 (48.2)	194 (38.1)	1122 (36.9)
≥ 15 kg gain	183 (16.0)	22 (22.9)	205 (16.5)	144 (18.3)	8 (20.0)	152 (18.3)	197 (30.8)	33 (37.1)	230 (31.6)	149 (32.9)	16 (28.6)	165 (32.4)	692 (22.8)

Numbers are means ± SD unless otherwise specified, * age 18 or 20 years

There was a suggestion for increased premenopausal breast cancer risk for taller *BRCA1* and *BRCA2* variant carriers, but the association was not significant (HR 1.19 per 10 cm increase, 95% CI 0.91–1.56 and HR 1.32, 95% CI 0.92–1.90, respectively). There was no evidence for an association with postmenopausal breast cancer (Table 4, Additional file 2: Table S2). HR estimates for young-adult BMI (continuous measure) were less than 1 for premenopausal breast cancer risk in both *BRCA1* and *BRCA2* variant carriers, although the associations were not statistically significant. In contrast, higher baseline BMI was associated with an increased risk for postmenopausal breast cancer for *BRCA1* variant carriers (HR per 5 kg/m^2^ increase 1.20, 95% CI 1.02–1.42). However, there was no association between higher baseline BMI and risk for *BRCA2* carriers (HR 0.97, 95% CI 0.81–1.17).

**Table 4:** Prospective analysis of height, body mass index, weight change, and breast cancer risk, by menopausal status

Menopausal status	Premenopausal								Postmenopausal							
BRCA status	*BRCA1*				*BRCA2*				*BRCA1*				*BRCA2*			
	n	BC	HR	95% CI	n	BC	HR	95% CI	n	BC	HR	95% CI	n	BC	HR	95% CI
Height, per 10 cm	1516	115	1.19	0.91–1.56	1011	51	1.32	0.92–1.90	905	112	0.97	0.73–1.30	626	67	0.95	0.68–1.31
Young-adult BMI per 5 kg/m^2^ (continuous)	1242	96	0.78	0.52–1.16	829	40	0.82	0.49–1.36	729	89	1.06	0.73–1.53	509	56	1.00	0.76–1.30
*Baseline BMI*, kg/m^2^ *(categories)*																
< 18.5	96	2	0.25	0.58– 1.08	33	1	0.71	0.11– 4.46	25	3	0.95	0.30–3.06	9	1	1.15	0.16–8.50
18.5 to < 25 (reference)	986	83	1.0		656	38	1.0		489	56	1.0		331	29	1.0	
≥25	434	30	0.89	0.57–1.39	322	12	0.59	0.30–1.15	391	53	1.21	0.81–1.82	286	37	1.13	0.68–1.89
Baseline BMI per 5 kg/m^2^ (continuous)	1516	115	1.00	0.84–1.20	1011	51	0.92	0.66–1.28	905	112	1.20	1.02–1.42	626	67	0.97	0.81–1.17
*Weight change (categories)*																
≥ 5 kg loss	93	6	0.76	0.34–1.75	52	2	1.40	0.23–8.65	28	4	1.67	0.61–4.61	29	6	2.53	0.97–6.58
< 5 kg loss to < 5 kg gain (reference)	506	34	1.0		324	11	1.0		183	18	1.0		121	7	1.0	
≥ 5 kg gain to < 15 kg gain	438	34	1.07	0.65–1.75	301	19	2.16	0.96–4.88	288	34	1.21	0.67–2.20	194	27	1.89	0.85–4.19
≥ 15 kg gain	205	22	1.43	0.80–2.54	152	8	1.14	0.46–2.82	230	33	1.63	0.88–3.04	165	16	1.18	0.46–3.05
Weight change*, per kg (continuous)	1242	96	1.04	0.94–1.16	829	40	1.07	0.92–1.24	729	89	1.10	1.01–1.19	509	56	0.99	0.88–1.11

All analyses were adjusted for age at menarche, number of full-term pregnancies, oral hormonal contraceptive use, and hormone replacement therapy

*BC* breast cancer, *BMI* body mass index, 1.0 = reference value

*Positive values mean weight gain and negative values mean weight loss

Between early adulthood and baseline premenopausal *BRCA1* variant carriers gained a mean of 6.5 ± 9.9 kg and postmenopausal *BRCA1* variant carriers gained 11.3 ± 11.9 kg. Similarly premenopausal *BRCA2* variant carriers gained a mean of 7.2 ± 10.5 kg and postmenopausal *BRCA2* variant carriers gained 11.4 ± 12.1 kg. In *BRCA1* variant carriers, weight gain was associated with increased risk of postmenopausal breast cancer (HR per kg gain 1.10, 95% CI 1.01–1.19); however, no significant association was found for *BRCA2* variant carriers (HR 0.99, 95% CI 0.88–1.11). For premenopausal breast cancer, no associations with weight change were observed in carriers of pathogenic variants in either gene.

These results were unchanged after adjustment for use of hormonal contraception and postmenopausal hormone therapy (Additional file 3: Table S3 and Additional file 4: Table S4). To find out if absolute weight was the better explanatory variable compared to BMI, we performed a sensitivity analysis and examined associations with baseline weight and young-adult weight separately. No large differences in association with breast cancer compared to baseline BMI and young-adult BMI were found (Additional file 5: Table S5). To question if the inverse association of young-adult BMI was related to lower height in adolescence or young adulthood, we performed an additional analysis of young-adult BMI, adjusting for height. The results remained similar (Additional file 5: Table S5).

## Discussion

In this large cohort of women carrying a pathogenic variant in *BRCA1* or *BRCA2*, we found associations of height, BMI, and weight gain with breast cancer risk in pre- and postmenopausal women, which were generally consistent in direction and magnitude with those described in the general population. This is the first study with a large prospective component to analyze the association anthropometric measures with breast cancer risk by menopausal status for *BRCA1* and *BRCA2* variant carriers separately.

Height as a risk factor for pre- and postmenopausal breast cancer has been described in prospective cohort studies, and in meta-analyses for the general population [1, 2]. We found positive associations for *BRCA2* variant carriers between height and risks of pre- and postmenopausal breast cancer in the retrospective analyses, and with premenopausal breast cancer risk in the prospective analysis; no associations with height were observed for *BRCA1* variant carriers, although the 95% CI include a 20% increased risk per 10 cm for all analyses except the retrospective analysis of premenopausal women. Height as a breast cancer risk factor for *BRCA2,* but not for *BRCA1* variant carriers, was also found in a retrospective study by Qian et al. [20]. The difference in the association between *BRCA1* and *BRCA2* carriers may be explained by the difference in proportions of estrogen receptor (ER)-positive breast cancer (22% for *BRCA1*, 77% for *BRCA2*) [27]. Zhang et al. showed, both in prospective studies and using Mendelian randomization, that height was a risk factor for hormone receptor positive breast cancer, and a weak or no association for receptor negative disease [2].

We found that higher young-adult BMI is associated with lower premenopausal breast cancer risk in both *BRCA1* and *BRCA2* variant carriers in the retrospective analysis, with consistent, although not statistically significant, risk estimates in the prospective analysis. This is in line with the associations seen in the general population, Mendelian randomization studies, and earlier retrospective studies by Manders et al. [17] and Qian et al. [20] for *BRCA1* and *BRCA2* variant carriers, the data of which partially overlapped with the retrospective data of the present study. The reasons for the inverse association of high young-adult BMI with breast cancer risk are unclear, but might be mediated through early breast tissue differentiation [4, 9]. In particular, childhood adiposity has been linked to a lower risk of benign breast disease and lower mammographic density which are risk factors for breast cancer [10, 11, 30]. Understanding the biological mechanism underlying the inverse association of higher young-adult BMI and lower premenopausal breast cancer risk could potentially identify modifiable pathways and provide new insights for prevention in the future.

Higher young-adult BMI is also associated with a lower risk of postmenopausal breast cancer in the general population [3, 5]. We found no association of higher young-adult BMI with postmenopausal breast cancer risk, which is also consistent with the findings by Qian et al. [20]. However, the retrospective cohort for postmenopausal women was much smaller than the premenopausal cohort and the HR estimates did not differ significantly.

In the prospective analysis of *BRCA1* pathogenic variant carriers, we found that higher baseline BMI and adult weight gain were associated with higher risk of postmenopausal breast cancer, but not with risk of premenopausal breast cancer. This is in line with the finding by Manders et al. for *BRCA1/2* variant carriers and with the findings in the general population [17, 31, 32]. However, for *BRCA2* variant carriers, we found no evidence of association, although a HR of similar magnitude compared to *BRCA1* variant carriers cannot be excluded, because of the wide confidence intervals.

A recent study has suggested that weight may be more predictive of postmenopausal breast cancer risk than BMI [33]. We analyzed the variables baseline weight and young-adult weight separately and found no large differences in association with breast cancer compared to baseline BMI and young-adult BMI (Additional file 4: Table S4).

Two biological explanations for our findings of high baseline BMI and of weight change and higher breast cancer risk in the postmenopausal cohort of carriers of *BRCA1* can be considered. A similar pattern (with an association for *BRCA1*- but not *BRCA2*-associated breast cancer) has been observed for oral contraceptive use [34]. Both findings were unexpected, since hormonal exposure is known to especially promote the ER-positive breast cancer subtype. However, in the general population, a weak association between elevated BMI and hormone receptor negative postmenopausal breast cancer and an association of oral contraception use with early onset triple negative breast cancer was reported [35-38]. One explanation for hormone-induced triple negative breast cancer might be the activation of the paracrine pathway via the receptor activator of nuclear factor kB (RANK) [39-46]. The pathway seems to be more relevant for carriers of variants in *BRCA1* compared to *BRCA2* or *PALB2* [47].

Early surgical menopause may lead to use of hormone therapy, usually a combination of estrogen and progestin. If BRCA1-deficient breast cells are particularly sensitive to hormonal treatment, this could lead to a significantly higher risk of breast cancer compared with *BRCA2* variant carriers associated with very early menopause and hormonal contraception use. In our study, however, the comparison of analysis with and without adjustment for use of hormonal contraception and postmenopausal hormone therapy did not support this hypothesis, since an association of high baseline BMI and weight gain with postmenopausal breast cancer risk was consistently seen in carriers of a pathogenic variant in *BRCA1,* but not in *BRCA2*.

Another mechanism of action promoting *BRCA1*-associated postmenopausal breast cancer may be the lack of suppression of aromatase. *BRCA1* is known to be an inhibitor of aromatase (CYP19) transcription and also interferes with transcriptional activation mediated by the estrogen receptor alpha (ER_α_) [47]. High BMI and weight gain might therefore lead to higher estradiol levels due to increased aromatase activity and consecutively to postmenopausal *BRCA1*-associated breast cancer. Interestingly, after menopause the percentage of ER-positive *BRCA1*-associated breast cancer increases [48].

Avoiding over- and underweight is important for both pathogenic variant carriers and non-carriers. Even if the relative risks for anthropometric measures and breast cancer in *BRCA1* and *BRCA2* variant carriers are similar to those found in the general population, the absolute excess risks will be higher for *BRCA1* and *BRCA2* variant carriers because of the higher background breast cancer risk. Additionally, as shown by Hopper et al., the postmenopausal elevation of breast cancer risk by far outweighs the premenopausal protection associated with high BMI [49].

Variant carriers may be highly motivated to attempt to modify their breast cancer risk through a healthy lifestyle [50, 51]. Healthy behavior change might include better nutrition and weight loss, although a persistent change in lifestyle is hard to achieve, especially after menopause [51]. Our retrospective results of low BMI and higher premenopausal breast cancer risk imply that underweight and unhealthy weight loss should be discouraged in premenopausal women. But further research on a larger dataset is necessary. The first intervention study on nutrition and physical activity for *BRCA1/2*-pathogenic variant carriers (the LIBRE trial in Germany) is currently recruiting [52]. It aims to show that physical fitness and maintaining a healthy body weight are key to good quality of life and—in the long run—prevent breast cancer in both affected and unaffected carriers.

Anthropometric measures are included in cancer risk prediction models such as BOADICEA [53]. In BOADICEA, the relative risks for height and BMI among variant carriers are assumed to be the same as those for the general population. The present analyses suggest that, for height, this is a reasonable assumption for *BRCA2,* but some adjustment may be needed for *BRCA1*. The association effect sizes for BMI are generally consistent with the general population estimates, at least for young-adult BMI [53].

The study has some limitations. Despite the large size of the cohort, the number of incident cases was still limited and the confidence limits were correspondingly wide, especially in subgroup analyses, such as postmenopausal high young-adult BMI. Thus, the results are still heavily dependent on the retrospective data, which are potentially subject to more bias. Much larger prospective cohorts with a broader age range and a longer follow-up are therefore needed.

A general challenge is the definition of menopausal status. Ideally, this would be defined by taking a detailed menstrual history and, in many cases, a blood test—but this is not feasible in large epidemiological studies, which rely on self-reporting. This may have led to some misclassification of postmenopausal women as premenopausal women, diluting the difference between the two groups.

A known limitation of retrospective studies is the potential for survival bias introduced by including prevalent cancer cases. There is evidence in the general population that higher BMI is associated with advanced tumor stage and a worse prognosis [54]. If the same is true for variant carriers, the inverse association with high BMI might be overestimated [55]. Some studies have addressed this by defining a pseudoincident cohort, which includes breast cancer cases only if they occurred within the last 5 years to avoid survival bias [34]. We did not utilize this approach, which would have substantially reduced the sample size. However, the retrospective and prospective findings were broadly consistent, though some associations (e.g., the inverse association with young-adult BMI in premenopausal women) were not statistically significant in the prospective analysis. Again, larger prospective analyses are needed.

This is the first prospective study to analyze the association of breast cancer risk with anthropometric measures by menopausal status, separately for *BRCA1* and *BRCA2* variant carriers. Height was a risk factor for premenopausal breast cancer in *BRCA2* variant carriers, but not *BRCA1* carriers. High young-adult BMI was associated with decreased risk of premenopausal breast cancer, but not for postmenopausal breast cancer. Higher BMI and weight gain in adult life were risk factors for postmenopausal breast cancer in *BRCA1* variant carriers.

In conclusion, our findings indicate that the associations of height, BMI and weight gain with breast cancer risk in *BRCA1* and *BRCA2* variant carriers are broadly similar to those reported in the general population when taking into account menopausal status. This research is not only important to inform carriers about age-specific cancer risks, it might also open up new risk reduction strategies by elucidating yet unknown signaling pathways.

## Supplementary Information

**Additional file 1: Table S1.** Overview of the studies contributing to the retrospective and prospective analyses.

**Additional file 2: Table S2.** Retrospective and prospective analysis of height in quintiles and breast cancer risk, by menopausal status.

**Additional file 3: Table S3.** Retrospective analysis of height, body mass index, weight change and breast cancer risk, by menopausal status.

**Additional file 4: Table S4.** Prospective analysis of associations between height, body mass index, weight change and breast cancer risk, by menopausal status.

**Additional file 5: Table S5.** Weight versus body mass index and breast cancer risk in *BRCA1* and *BRCA2* variant carriers, by menopausal status.

## Data Availability

The dataset supporting the conclusions of this article is available upon reasonable request. Requests should be made to Dr. Karin Kast (University Hospital Cologne, karin.kast@uk-koeln.de).
