# Supplementary material for: Associations of height, body mass index, and weight gain with breast cancer risk in carriers of a pathogenic variant in BRCA1 or BRCA2: the BRCA1 and BRCA2 Cohort Consortium
Source: Breast Cancer Res. 2023 Jun 20;25:72. doi: 10.1186/s13058-023-01673-w (PMC10280955; doi:10.1186/s13058-023-01673-w)
Supplement: Supplementary file 1 — Additional file 1: Table S1. Overview of the studies contributing to the retrospective and prospective analyses. [file 13058_2023_1673_MOESM1_ESM.docx]

**Additional file 1. Overview of the studies contributing to the retrospective and prospective analyses**

|  | Retrospective | | | Prospective | | | Combined |
| --- | --- | --- | --- | --- | --- | --- | --- |
| STUDY | *BRCA1* | *BRCA2* | total | *BRCA1* | *BRCA2* | total | *BRCA1/BRCA2* |
| BCFR | 0 | 0 | 0 | 292 | 217 | 509 | 509 |
| Belgium | 3 | 0 | 3 | 0 | 0 | 0 | 3 |
| CNIO ES | 36 | 42 | 78 | 6 | 6 | 12 | 78 |
| DKFZ /Düsseldorf DE | 7 | 0 | 7 | 0 | 0 | 0 | 7 |
| EMBRACE | 1455 | 1294 | 2749 | 458 | 470 | 928 | 2777 |
| GC–HBOC | 119 | 70 | 189 | 29 | 15 | 44 | 191 |
| GENEPSO | 931 | 571 | 1502 | 475 | 322 | 797 | 1532 |
| HEBON | 613 | 174 | 787 | 233 | 70 | 303 | 843 |
| HSP ES | 2 | 0 | 2 | 0 | 0 | 0 | 2 |
| INHERIT | 63 | 74 | 137 | 29 | 31 | 60 | 137 |
| kConFab | 0 | 0 | 0 | 314 | 278 | 592 | 592 |
| LUND–BRCA | 81 | 25 | 106 | 44 | 13 | 57 | 117 |
| Milan IT | 33 | 11 | 44 | 0 | 0 | 0 | 44 |
| MMCI CZ | 225 | 80 | 305 | 83 | 28 | 111 | 305 |
| MSC ES | 80 | 62 | 142 | 31 | 22 | 53 | 143 |
| MUV–Austria | 252 | 96 | 348 | 63 | 19 | 82 | 349 |
| NIO–Hungary | 95 | 30 | 125 | 25 | 11 | 36 | 125 |
| OUH | 100 | 59 | 159 | 45 | 27 | 72 | 161 |
| Poland | 91 | 0 | 91 | 27 | 0 | 27 | 92 |
| STOCKHOLM–BRCA | 71 | 13 | 84 | 13 | 2 | 15 | 84 |
| Total | 4257 | 2601 | 6858 | 2167 | 1531 | 3698 | 8091 |
