# Supplementary material for: Associations of height, body mass index, and weight gain with breast cancer risk in carriers of a pathogenic variant in BRCA1 or BRCA2: the BRCA1 and BRCA2 Cohort Consortium
Source: Breast Cancer Res. 2023 Jun 20;25:72. doi: 10.1186/s13058-023-01673-w (PMC10280955; doi:10.1186/s13058-023-01673-w)
Supplement: Supplementary file 2 — Additional file 2: Table S2. Retrospective and prospective analysis of height in quintiles and breast cancer risk, by menopausal status. [file 13058_2023_1673_MOESM2_ESM.docx]

**Additional file 2: Retrospective and prospective analysis of height in quintiles and breast cancer risk by menopausal status.**

| Menopausal status | | | Premenopausal | | | | | | | | Postmenopausal | | | | | | | |
| --- | --- | --- | --- | --- | --- | --- | --- | --- | --- | --- | --- | --- | --- | --- | --- | --- | --- | --- |
| BRCA status | | | *BRCA1* | | | | *BRCA2* | | | | *BRCA1* | | | | *BRCA2* | | | |
|  | | | n | BC | HR | 95%CI | n | BC | HR | 95%CI | n | BC | HR | 95%CI | n | BC | HR | 95%CI |
| Height in Quintiles Q1-Q5 | Retrospective | Q1 (128 – 159 cm), reference | 856 | 324 | 1.0 |  | 578 | 175 | 1.0 |  | 178 | 68 | 1.0 |  | 154 | 52 | 1.0 |  |
|  |  | Q2 (160 – 163 cm) | 1004 | 346 | 0.97 | 0.80 – 1.17 | 652 | 220 | 1.30 | 1.00 – 1.68 | 177 | 58 | 1.21 | 0.82 – 1.8 | 136 | 55 | 1.70 | 1.05 – 2.76 |
|  |  | Q3 (164 – 167 cm) | 795 | 295 | 1.10 | 0.89 – 1.36 | 399 | 119 | 0.97 | 0.71 – 1.33 | 114 | 32 | 1.04 | 0.64 – 1.69 | 74 | 24 | 1.74 | 0.97 – 3.15 |
|  |  | Q4 (168 – 170 cm) | 770 | 291 | 1.13 | 0.92 – 1.40 | 484 | 151 | 1.16 | 0.87 – 1.54 | 89 | 21 | 0.68 | 0.35 – 1.34 | 91 | 36 | 1.63 | 0.92 – 2.91 |
|  |  | Q5 (171 – 196 cm) | 832 | 272 | 0.98 | 0.78 – 1.22 | 488 | 171 | 1.53 | 1.13 – 2.05 | 92 | 20 | 0.60 | 0.35 – 1.06 | 51 | 12 | 1.34 | 0.61 – 2.94 |
|  | Prospective | Q1 (124 – 160 cm), reference | 359 | 25 | 1.0 |  | 266 | 11 | 1.0 |  | 275 | 39 | 1.0 |  | 215 | 24 | 1.0 |  |
|  |  | Q2 (161 – 163 cm) | 220 | 19 | 1.43 | 0.77 – 2.66 | 153 | 9 | 1.52 | 0.61 – 3.78 | 149 | 21 | 0.98 | 0.54 – 1.75 | 110 | 14 | 0.92 | 0.46 – 1.84 |
|  |  | Q3 (164 – 167 cm) | 265 | 23 | 1.23 | 0.71 – 2.15 | 181 | 7 | 1.34 | 0.48 – 3.74 | 179 | 17 | 0.61 | 0.33 – 1.13 | 114 | 12 | 0.86 | 0.44 – 1.69 |
|  |  | Q4 (168 – 170 cm) | 276 | 21 | 1.28 | 0.71 – 2.30 | 210 | 12 | 2.42 | 1.00 – 5.85 | 155 | 13 | 0.55 | 0.27 – 1.11 | 109 | 13 | 1.22 | 0.61 – 2.44 |
|  |  | Q5 (171 – 188 cm) | 306 | 27 | 1.27 | 0.72 – 2.24 | 201 | 12 | 1.52 | 0.64 – 3.62 | 147 | 22 | 0.94 | 0.52 – 1.69 | 78 | 4 | 0.64 | 0.23 – 1.83 |

Legend: BC=breast cancer, 1.0 = reference value

Analysis with adjustments for age at menarche, number of full term pregnancies, oral hormonal contraceptive use and hormone replacement therapy.
