# Supplementary material for: Associations of height, body mass index, and weight gain with breast cancer risk in carriers of a pathogenic variant in BRCA1 or BRCA2: the BRCA1 and BRCA2 Cohort Consortium
Source: Breast Cancer Res. 2023 Jun 20;25:72. doi: 10.1186/s13058-023-01673-w (PMC10280955; doi:10.1186/s13058-023-01673-w)
Supplement: Supplementary file 3 — Additional file 3: Table S3. Retrospective analysis of height, body mass index, weight change and breast cancer risk, by menopausal status. [file 13058_2023_1673_MOESM3_ESM.docx]

**Additional file 3: Retrospective analysis of height, body mass index, weight change and breast cancer risk, by menopausal status**

| Menopausal status | | Premenopausal | | | | | | | | Postmenopausal | | | | | | | |
| --- | --- | --- | --- | --- | --- | --- | --- | --- | --- | --- | --- | --- | --- | --- | --- | --- | --- |
| BRCA status | | *BRCA1* | | | | *BRCA2* | | | | *BRCA1* | | | | *BRCA2* | | | |
|  | | n | BC | HR | 95%CI | n | BC | HR | 95%CI | n | BC | HR | 95%CI | n | BC | HR | 95%CI |
| Height, per 10 cm | | 4424 | 1583 | 1.06 | 0.97–1.17 | 2686 | 865 | 1.17 | 1.02–1.35 | 670 | 205 | 0.83 | 0.66–1.05 | 526 | 187 | 1.36 | 1.02–1.81 |
| Young–adult BMI, kg/m² (categories) | <18.5 | 737 | 277 | 1.12 | 0.95–1.32 | 375 | 138 | 1.18 | 0.92–1.51 | 95 | 33 | 1.55 | 1.03–2.34 | 66 | 33 | 1.62 | 0.99–2.65 |
|  | 18.5–< 25 | 3364 | 1219 | 1.0 |  | 2082 | 677 | 1.0 |  | 520 | 153 | 1.0 |  | 410 | 140 | 1.0 |  |
|  | >=25 | 323 | 87 | 0.71 | 0.55–0.92 | 229 | 50 | 0.69 | 0.49–0.97 | 55 | 19 | 1.23 | 0.73–2.04 | 50 | 14 | 0.88 | 0.49–1.59 |
| Young–adult BMI per 5 kg/m^2^ (continuous) | | 4424 | 1583 | 0.79 | 0.70–0.88 | 2686 | 865 | 0.76 | 0.66–0.88 | 670 | 205 | 0.95 | 0.71–1.27 | 526 | 187 | 0.81 | 0.60–1.10 |

Legend: BC=breast cancer, BMI=body mass index, 1.0=reference value

Retrospective analysis without adjustments for age at menarche, number of full term pregnancies, oral hormonal contraceptive use and hormone replacement therapy.
