# Supplementary material for: Associations of height, body mass index, and weight gain with breast cancer risk in carriers of a pathogenic variant in BRCA1 or BRCA2: the BRCA1 and BRCA2 Cohort Consortium
Source: Breast Cancer Res. 2023 Jun 20;25:72. doi: 10.1186/s13058-023-01673-w (PMC10280955; doi:10.1186/s13058-023-01673-w)
Supplement: Supplementary file 4 — Additional file 4: Table S4. Prospective analysis of associations between height, body mass index, weight change and breast cancer risk, by menopausal status. [file 13058_2023_1673_MOESM4_ESM.docx]

**Aditional file 4: Prospective analysis of associations between height, body mass index, weight change and breast cancer risk, by menopausal status**

| Menopausal status | | Premenopausal | | | | | | | | Postmenopausal | | | | | | | |
| --- | --- | --- | --- | --- | --- | --- | --- | --- | --- | --- | --- | --- | --- | --- | --- | --- | --- |
| BRCA status | | *BRCA1* | | | | *BRCA2* | | | | *BRCA1* | | | | *BRCA2* | | | |
|  | | n | BC | HR | 95%CI | n | BC | HR | 95%CI | n | BC | HR | 95%CI | n | BC | HR | 95%CI |
| Height, per 10 cm | | 1549 | 117 | 1.19 | 0.91–1.55 | 1035 | 51 | 1.41 | 1.00–1.98 | 935 | 119 | 0.95 | 0.72–1.25 | 645 | 72 | 0.95 | 0.69–1.31 |
| Young–adult BMI per 5 kg/m^2^ (continuous) | | 1264 | 97 | 0.81 | 0.56–1.17 | 845 | 40 | 0.75 | 0.48–1.17 | 749 | 94 | 1.10 | 0.78–1.55 | 519 | 58 | 1.01 | 0.78–1.32 |
| Baseline BMI, kg/m² (categories) | <18.5 | 97 | 2 | 0.25 | 0.06–1.05 | 33 | 1 | 0.76 | 0.11–5.04 | 25 | 3 | 0.94 | 0.29–3.01 | 9 | 1 | 1.07 | 0.15–7.67 |
|  | 18.5–< 25 | 1009 | 85 | 1.0 |  | 673 | 38 | 1.0 |  | 506 | 59 | 1.0 |  | 344 | 32 | 1.0 |  |
|  | >=25 | 443 | 30 | 0.86 | 0.56–1.33 | 329 | 12 | 0.52 | 0.29–0.95 | 404 | 57 | 1.24 | 0.85–1.82 | 292 | 39 | 1.16 | 0.72–1.88 |
| Baseline BMI per 5 kg/m^2^(continuous) | | 1549 | 117 | 0.99 | 0.83–1.19 | 1035 | 51 | 0.83 | 0.61–1.13 | 935 | 119 | 1.19 | 1.02–1.39 | 645 | 72 | 0.99 | 0.84–1.18 |
| Weight change, per kg | ≥5 kg loss | 95 | 6 | 0.79 | 0.35–1.80 | 53 | 2 | 1.27 | 0.29–5.61 | 29 | 4 | 1.62 | 0.58–4.48 | 29 | 6 | 2.42 | 0.96–6.10 |
|  | <5 kg loss to <5 kg gain (reference) | 513 | 34 | 1.0 |  | 330 | 11 | 1.0 |  | 185 | 19 | 1.0 |  | 125 | 8 | 1.0 |  |
|  | ≥5 kg gain to <15 kg gain | 499 | 35 | 1.09 | 0.67–1.78 | 308 | 19 | 1.82 | 0.82–4.03 | 299 | 37 | 1.23 | 0.69–2.17 | 198 | 27 | 1.76 | 0.84–3.70 |
|  | ≥15 kg gain | 207 | 22 | 1.44 | 0.81–2.54 | 154 | 8 | 0.96 | 0.40–2.32 | 236 | 34 | 1.56 | 0.84–2.87 | 167 | 17 | 1.11 | 0.46–2.66 |
|  | ≥5 kg loss | 1264 | 97 | 1.04 | 0.95–1.15 | 845 | 40 | 1.02 | 0.89–1.16 | 749 | 94 | 1.09 | 1.01–1.18 | 519 | 58 | 0.99 | 0.89–1.10 |

Legend: BC=breast cancer, BMI=body mass index, 1.0=reference value

Prospective analysis without adjustments for age at menarche, number of full term pregnancies, oral hormonal contraceptive use and hormone replacement therapy.
