# Supplementary material for: Associations of height, body mass index, and weight gain with breast cancer risk in carriers of a pathogenic variant in BRCA1 or BRCA2: the BRCA1 and BRCA2 Cohort Consortium
Source: Breast Cancer Res. 2023 Jun 20;25:72. doi: 10.1186/s13058-023-01673-w (PMC10280955; doi:10.1186/s13058-023-01673-w)
Supplement: Supplementary file 5 — Additional file 5: Table S5. Weight versus body mass index and breast cancer risk in BRCA1 and BRCA2 variant carriers, by menopausal status. [file 13058_2023_1673_MOESM5_ESM.docx]

**Additional file 5: Weight versus body mass index and breast cancer risk in *BRCA1* and *BRCA2* variant carriers, by menopausal status**

| **Premenopausal** | | | | | | | | | **Postmenopausal** | | | | | | | |
| --- | --- | --- | --- | --- | --- | --- | --- | --- | --- | --- | --- | --- | --- | --- | --- | --- |
|  | ***BRCA1*** | | | | ***BRCA2*** | | | | ***BRCA1*** | | | | ***BRCA2*** | | | |
|  | n | BC | HR | 95%CI | n | BC | HR | 95%CI | n | BC | HR | 95%CI | n | BC | HR | 95%CI |
|  |  |  |  |  |  |  |  |  |  |  |  |  |  |  |  |  |
| **Retrospective** | |  |  |  |  |  |  |  |  |  |  |  |  |  |  |  |
| Young–adult weight (per 5 kg) | 4257 | 1528 | 0.93 | 0.89–0.97 | 2601 | 836 | 0.95 | 0.91–1.00 | 650 | 199 | 0.92 | 0.81–1.03 | 506 | 179 | 0.99 | 0.90–1.08 |
| Young–adult BMI (per 5 kg/m^2^) | 4257 | 1528 | 0.74 | 0.66–0.84 | 2601 | 836 | 0.76 | 0.65–0.89 | 650 | 199 | 0.86 | 0.64–1.17 | 506 | 179 | 0.85 | 0.64–1.14 |
| Young–adult BMI (per 5 kg/m^2^) * | 4257 | 1528 | 0.75 | 0.66–0.85 | 2601 | 836 | 0.77 | 0.66–0.90 | 650 | 199 | 0.83 | 0.61–1.14 | 506 | 179 | 0.85 | 0.64–1.13 |
| **Prospective** | | |  |  |  |  |  |  |  | |  |  |  |  |  |  |
| Young–adult weight (per 5 kg) | 1242 | 96 | 0.98 | 0.87–1.09 | 829 | 40 | 0.99 | 0.85–1.16 | 729 | 89 | 1.00 | 0.89–1.13 | 509 | 56 | 1.00 | 0.90–1.10 |
| Young–adult BMI (per 5 kg/m^2^) | 1242 | 96 | 0.78 | 0.52–1.16 | 829 | 40 | 0.82 | 0.49–1.36 | 729 | 89 | 1.06 | 0.73–1.53 | 509 | 56 | 1.00 | 0.77–1.30 |
| Young–adult BMI (per 5 kg/m^2^)* | 1242 | 96 | 0.80 | 0.54–1.20 | 829 | 40 | 0.85 | 0.51–1.41 | 729 | 89 | 1.05 | 0.73–1.52 | 509 | 56 | 1.00 | 0.76–1.30 |
| Baseline weight (per 5 kg) | 1516 | 115 | 1.02 | 0.96–1.07 | 1011 | 51 | 1.01 | 0.90–1.13 | 905 | 112 | 1.06 | 1.00–1.12 | 626 | 67 | 0.99 | 0.92–1.06 |
| Baseline BMI (per 5 kg/m^2^) | 1516 | 115 | 1.00 | 0.84–1.2 | 1011 | 51 | 0.92 | 0.66–1.28 | 905 | 112 | 1.20 | 1.02–1.42 | 626 | 67 | 0.97 | 0.81–1.17 |

Legend: BC=breast cancer, BMI=body mass index

All analyses were adjusted for age at menarche, number of full term pregnancies, oral hormonal contraceptive use and hormone replacement therapy.

* additionally adjusted for height
